# Chitinase-functionalized UiO-66 framework nanoparticles active against multidrug-resistant *Candida Auris*

**DOI:** 10.1186/s12866-024-03414-1

**Published:** 2024-07-20

**Authors:** Shaymaa A. Ismail, Bahgat Fayed, Reda M. Abdelhameed, Amira A. Hassan

**Affiliations:** 1 Department of Chemistry of Natural and Microbial Products, Pharmaceutical and Drug Industries Research Institute, National Research Centre, P.O. 12622, 33 El Bohouth Street, Dokki, Giza, Egypt; 2 Applied Organic Chemistry Department, Chemical Industries Research Institute, National Research Centre, 33 EL Buhouth St, Dokki, Giza 12622 Egypt

**Keywords:** Chitinase, *Candida auris*, UiO66, *Talaromyces varians*, SSW3

## Abstract

*Candida auris* (*C. auris*) is a yeast that has caused several outbreaks in the last decade. Cell wall chitin plays a primary role in the antifungal resistance of *C. auris*. Herein, we investigated the potential of chitinase immobilized with UiO-66 to act as a potent antifungal agent against *C. auris*. Chitinase was produced from *Talaromyces varians* SSW3 in a yield of 8.97 U/g dry substrate (ds). The yield was statistically enhanced to 120.41 U/g ds by using Plackett–Burman and Box–Behnken design. We synthesized a UiO-66 framework that was characterized by SEM, TEM, XRD, FTIR, a particle size analyzer, and a zeta sizer. The produced framework had a size of 70.42 ± 8.43 nm with a uniform cubic shape and smooth surface. The produced chitinase was immobilized on UiO-66 with an immobilization yield of 65% achieved after a 6 h loading period. The immobilization of UiO-66 increased the enzyme activity and stability, as indicated by the obtained K_d_ and T_1/2_ values. Furthermore, the hydrolytic activity of chitinase was enhanced after immobilization on UiO-66, with an increase in the V_max_ and a decrease in the K_m_ of 2- and 38-fold, respectively. Interestingly, the antifungal activity of the produced chitinase was boosted against *C. auris* by loading the enzyme on UiO-66, with an MIC_50_ of 0.89 ± 0.056 U/mL, compared to 5.582 ± 0.57 U/mL for the free enzyme. This study offers a novel promising alternative approach to combat the new emerging pathogen *C. auris.*

**Supplementary Information:**

The online version contains supplementary material available at 10.1186/s12866-024-03414-1.

## Introduction

Chitin is one of the most widespread biopolymer on earth. It is a hydrophobic water insoluble polymer composed of linear chains of repeating units of β (1,4)-N-acetylglucosamine connected via β-1,4-glycosidic linkages with annual production exceeding 1000 billion tons [1, 2]. It is considered a crucial part of the fungal cell wall that compromises 3 to 25% of the fungal biomass [3]. The variation of chitin in the fungal cell wall is species-specific variation and also vary among different growth stages within a single species. For instance, higher fungi such as Ascomycetes and Basidiomycetes typically have lower chitin content compared to lower fungi like Zygomycetes [4]. Further, chitin content might be higher during spore formation and lower during active vegetative growth [5].

Although chitin possesses unique characteristics, such as bio-compatibility, bio-degradability, low immunogenicity, and wide availability, which are suitable criteria for several pharmaceutical, packaging, and biomedical food applications, its insolubility in water and most organic solvents limits the ability of large volumes of chitin-based products to enter the market [6]. Chitinases (EC 3.2.2.14) are glycosyl hydrolytic enzymes that degrade chitin by hydrolyzing the β-1,4 linkages of N-acetyl glucosamine units. These enzymes are classified according to their site of action into endo- and exo-acting enzymes. Endochitinases cleave chitin along the internal chain at random positions, while exochitinases cleave chitin at the terminal end [7]. Consequently, the degradation of chitin yields either N-acetyl glucosamine and/or longer oligosaccharides with variable biological applications [8]. Chitinases are produced by organisms such as fungi and insects, and they can also be detected in bacteria and higher plants [9]. In general, microorganisms use chitinases to remodel their chitin content or to parasitize other chitin-containing organisms to digest the chitin layers into absorbable metabolites as a nitrogen and carbon energy source [3, 8, 10–12]. Production of chitinases from microorganism for biological application has been explored by different studies [13–16]. Optimizing the enzyme production from microorganism is crucial for industrial application. The combined application of Plackett–Burman and Box–Behnken designs provides a robust framework for optimizing enzyme production from microorganisms [17]. Initially, the Plackett–Burman design is used to screen and identify the most significant factors. Subsequently, the Box–Behnken design fine-tunes these factors, enabling precise optimization of the production process. This systematic approach ensures that all critical variables are considered, interactions are thoroughly examined, and the enzyme production process is optimized for maximum efficiency and yield [18, 19].

There are numerous industrial applications of chitinases. For instance, it can be used to treat and manage waste from the seafood industry. Biological control of pathogenic fungi, insects, and pests is another important application for chitinases. Furthermore, chitin degradation products can be exploited in the biomedical sector, and N-acetyl glucosamine can be applied in the treatment and prevention of various inflammatory disorders. Additionally, chitooligomers can be used as prebiotic supplements and as antitumor, antioxidant, anti-inflammatory, hypocholesterolemic and immunomodulatory agents [20–26].

Among the several applications of chitinases, the biological control of pathogenic fungi and insects is gaining popularity in the agricultural and medical sectors. For agricultural application, Zarei et al., [15] produced chitinase from *Serratia marcescens B4A* that exhibited potent antifungal activity against *Alternaria brassicicola*,* Alternaria raphani*,* Bipolaris* sp. and *Rhizoctonia solani.* Recombinant chitinase heterologously expressed into the *BL21 (DE3) Escherichia coli* strain showed antifungal activity against *Alternaria brassicicola*,* Alternaria raphani*,* Bipolaris* sp., *Botrytis cinerea*,* Fusarium graminearum*,* Rhizoctonia solani*,* Sclerotinia sclerotiorum*, and *Trichoderma reesei* [27]. Matroodi and her colleagues fused the chitinase Chit42 from *Trichoderma atroviride* PTCC5220 with the ChBD from *Serratia marcescens* chitinase B to produce a chimeric chitinase with potent chitin-binding capacity [28]. The chimeric chitinase demonstrated antifungal activity against *Alternaria alternata*,* Rhizoctonia solani*, and *Sclerotinia sclerotiorum.*

Apart from combating fungal infection for agricultural applications, chitinase is considered a promising tool for controlling human fungal infection. Invasive fungal infection in humans is a major concern for people with altered immune function owing to the limited numbers of effective antifungal drugs on the market and the emergence of drug-resistant fungal forms [29, 30]. While healthy individuals combat fungal infection via the innate immune system and primarily via pulmonary defense mechanisms, immunocompromised patients are susceptible to fungal infection, particularly in intensive care settings [31]. The fungal cell wall consists of a rigid layer of chitin in addition to β-glucans and mannoproteins [32]. Hence, chitinase can lyse fungal cells by degrading the chitin content of the cell wall. For instance, recombinant human chitinase inhibited the fungal pathogen *Cryptococcus neoformans* growth in vitro and increased the survival of mice infected with *C. albicans* by degrading the fungal cell wall [33, 34]. Furthermore, chitinase obtained from *Lactobacillus rhamnosus* GG inhibited *C. albicans* hyphal morphogenesis in vitro [35]. Additionally, Abdul-Ghani and his colleagues showed that the combination of zinc oxide nanoparticles and chitinase enzymes exhibited potent antifungal activity against *Fusarium* species [36]. Given the potent antifungal activity of chitinase enzymes, their application becomes particularly relevant in the context of emerging fungal threats, notably *Candida auris* (*C. auris*) infections. In the last decade, *C. auris has* emerged as a resistant yeast that causes serious global health threats for patients in healthcare facilities [37–39]. Since the initial detection of *C. auris* in 2009, it has gained much attention with a recent alert from the Centers for Disease Control and Prevention (CDC) due to its high infectivity. The resulting yeast demonstrated a high resistance profile against commonly used antifungal drugs with improved transmission dynamics and caused severe infections in the skin, ears, wounds, and bloodstream [40]. During the COVID-19 epidemic, outbreaks of *C. auris* infection dramatically increased according to CDC [41]. Reports have highlighted that the prevalence of *C. auris* infection was approximately 23% among patients infected with COVID-19 who stayed in the ICU for more than 21 days [42]. Caspofungin is the first treatment for *C. auris* infection; however, recent studies have shown the emergence of caspofungin resistance among *C. auris* clinical isolates [43–46]. Additionally, caspofungin-susceptible *C. auris* can later acquire resistance by modifying the cell wall chitin [32]. The pronounced increase in *C. auris* resistance to caspofungin, in addition to its high mortality rate, urges the identification of new tools to combat these emerging resistant strains [47]. This study, for the first time to our knowledge, aims to develop innovative tools to combat *C. auris* by utilizing a microbial-produced chitinase enzyme incorporated on a metal-organic framework. The chitinase enzyme can degrade the chitin in the *Candida* cell wall, while the metal-organic framework enhances the enzyme’s stability and improves cellular uptake. To better understand the mechanism behind this approach, it is important to explore the nature of metal-organic frameworks. Metal-organic frameworks are hybrid materials consisting of metal-based clusters formed in three dimensions by employing various organic linkers [48]. UiO-66 is an archetypal metal-organic framework composed of [Zr_6_O_4_(OH)_4_] clusters with 1,4-benzodicarboxylic acid struts. It is thermally stable up to 350 °C owing to its strong Zr-O bond and very large surface area [49].

Its exceptional uniform pore size, water solubility, and flexibility of selecting the building units according to the nature of the cargo, in addition to being biocompatible, allow the loading of pharmaceutical cargo and efficient cellular penetration for drug delivery applications [48–51]. For instance, UiO-66 prepared by a solvothermal route was successfully loaded with tramadol for oral drug delivery [52]. Furthermore, Jarai et al., used UiO-66 nanoparticles as an aerosol platform for pulmonary drug delivery applications [48]. While several studies have provided in-depth information about the potential of UiO-66 nanoparticles to act as carriers for pharmaceutical drugs and some enzymes, there is a major lack of understanding of the ability of UiO-66 nanoparticles to deliver and augment the antifungal activity of bioactive enzymes. We hypothesize that UiO-66 nanoparticles can enhance the antifungal effectiveness of chitinase by providing a protective shielding for the enzyme. This shielding enhances chitinase’s resilience across diverse temperature and pH ranges, safeguarding it against proteolytic degradation and other environmental factors that might otherwise deactivate it. Moreover, the porous framework of UiO-66 aids in preserving the optimal orientation and structure of chitinase, thereby amplifying its enzymatic activity. Additionally, the nanoparticles has massive surface area, facilitating heightened chitinase loading, which ensures a more concentrated presence of active enzyme in the vicinity of *C. auris*.

In our current research, our objective was to develop a novel approach for addressing *C. auris* by creating UiO-66 nanoparticles loaded with chitinase enzyme. The chitinase enzyme was produced from Talaromyces varians SSW3. We optimized chitinase production using Plackett–Burman and Box–Behnken designs. Subsequently, we immobilized the produced enzyme onto UiO-66 nanoparticles. We then evaluated the antifungal efficacy of these nanoparticles loaded with chitinase against *C. auris*.

## Materials and methods

### Materials

Shrimp-chitin, N-acetyl glucosamine, 4-nitrophenylN-acetyl-β-D-glucosaminide, 4-nitrophenol, and dinitrosalicylic acid (DNS) were purchased from Sigma‒Aldrich, Saint Louis, USA. N-Acetyl oligosaccharides (chitopentose and chitohexose) were obtained from Seikagaku Biobusiness Corporation, Tokyo, Japan. Potato dextrose agar (PDA) and silica gel 60 thin layer chromatography (TLC) plates were purchased from Merck, Darmstadt, Germany. Wheat bran was purchased from a local market. Shrimp-byproducts (cephalothoraxes and carapaces) were obtained from the local seafood market and then processed according to Benhabiles et al. [53].

### Microorganisms and fermentation conditions

The examined fungus in the present study was isolated at the chemistry of the Natural and Microbial Product Department of the National Research Center, Dokki, Giza, Egypt. It was initially cultured on PDA and maintained at 30 °C for 7 days before further use. Identification of the isolate was carried out depending on the culture, microscopic features, and the 18 S rDNA sequencing performed by Sigma Scientific Services Co.

Enzyme production was carried out via solid-state fermentation (SSF) of 1 g of shrimp-byproducts added to 4 g of wheat bran and moistened with 10 mL of moistening solution as described by Ismail et al. [54]. The moistening solution was composed of (g%) K_2_HPO_4_ (0.15%), KCl (0.2%), MgSO_4_ (0.01%), FeSO4·7H2O (0.01%) and yeast extract (1.85%). Initially, a 7-day-old PDA slant was used for the cultivation of 50 mL of an inoculum consisted of (g %): yeast extract, 0.05; MgSO_4_.7H_2_O, 0.05 KH_2_PO_4_, 0.1; peptone, 0.5 and dextrose, 2, followed by incubation at 30 °C for 48 h. After that, 100% (v/w) of the inoculum was used for the cultivation of the fermentation medium and then incubated at 30 °C for 5 days. After incubation, the fermented substrate was extracted using 50 mL of distilled water at 30 °C and 180 rpm for 1 h and then centrifuged at 4000 rpm for 10 min and the clear supernatant was used furtherly.

### Enzyme activity and protein content

The chitinase activity was determined as described by Rustiguel et al. [55] using 0.1% of the synthetic substrate 4-nitrophenyl N-acetyl-β-D-glucosaminide, in which equivalent volumes (50 µL each) of the enzyme and the substrate was incubated at 50 °C for 15 min and at the end of the incubation period, 1 mL of 1 M NaOH was added and the optical density of the developed color was estimated at 410 nm. One unit of the enzyme was the amount of the enzyme capable of releasing 1 µmol of p-nitrophenol/ min (equivalent to 1 µmol of N-acetyl glucosamine) using a standard curve of p-nitrophenol. The protein content was estimated as described by Lowry et al. [56] using standard bovine serum albumin. According to Lowry method, 5 mL of Lowry reagent was added to 1 mL of the suitable dilution of the sample and left to stand for 10 min at room temperature then 0.5 mL of diluted Folin reagent was added and after 20 min, the optical density of the produced color was estimated at 750 nm.

### Optimization process

The described fermentation medium was optimized by examining single variable-at-a time followed by statistical optimization.

#### Single variable-at-a time optimization

Initially, the effect of the presence of each salt in the fermentation medium on the enzyme productivity was evaluated by the individual removal of each salt. After that, the effect of different shrimp-byproduct-to-wheat bran ratios on enzyme productivity was evaluated using the described fermentation medium as the control. This was followed by the examination of the addition of different concentrations of yeast extract on the moistening agent using the optimized medium in the previous step as the control.

#### Statistical optimization

Fermentation conditions was optimized on the base of two sequential steps. First, the variables that had significant impact on the enzyme productivity were estimated via the application of Plackett–Burman design, and then the selected variables were optimized via the application of Box–Behnken design [57, 58].

#### Plackett–Burman design

The screening of seven variables (initial moisture content, K_2_HPO_4_ concentration, KCl concentration, moistening agent pH, inoculum size, temperature and fermentation period) was carried out in eight experimental runs in which the estimated response was the enzyme activity. The effect of each variable was evaluated at two levels, high (+ 1) and low (-1) (Supplementary Table 1), and then calculated according to Eq. (1).

1 $${E_{(Xi)}} = {\text{ }}2\left( {\Sigma {\text{ }}{M_{i + }} - {M_{i - }}} \right)/N$$

where E_(Xi)_ is the impact of the examined variable. M_i+_ and M_i−_ are the observed enzyme activity, where the examined variable (Xi) is adjusted at high and low values, respectively, and N is the number of trials.

#### Box‒Behnken design

The variables with the greatest significant effect on the enzyme productivity (inoculum size, temperature, and incubation period) were optimized via the application of Box–Behnken design, in which each variable was examined at 3 levels (low (-), high (+) and control (0)) in 15 experimental runs with 3 central points as illustrated in supplementary Table (3). The correlation between the examined variables and the activity of the enzyme was represented on the basis of the following second-order polynomial equation:

2 $$Y = {\text{ }}{B_0} + \Sigma {\text{ }}{B_i}{X_i} + \Sigma {\text{ }}{B_{ii}}{X_i}^2 + \Sigma {\text{ }}{B_{ij}}{X_i}{X_j}$$

where Y is the predicted enzyme activity; ß_0_ is the intercept of the model; ß_i_ is the linear coefficient; ß_ii_ is the quadratic coefficient; and ß_ij_ is the cross-product coefficient, where X_i_ and X_j_ are the coded levels of the examined variables.

### Enzyme precipitation

The clear supernatant that possessed the highest activity was precipitated using ethanol and acetone at concentrations ranging from 30 to 90% at 10% intervals. After that, the specific activity of each fraction was calculated on the base of the estimated enzyme activity and protein content [59].

### Hydrolytic activity of the produced enzyme

In order to elucidate whether the produced enzyme possessed exo- or endo-hydrolytic activity, chitin oligosaccharide hydrolyzed products were estimated. Initially, the enzyme hydrolytic activity was estimated by evaluating its efficiency in the hydrolysis of a 0.1% solution of chitin oligosaccharide (N-acetyl chitopentose and N-acetyl chitohexose) via the addition of equivalent volume of the enzyme with activity 2 U/mL and incubated at 50 °C for 1 h. After that, the hydrolysate was analyzed by TLC, in which the mobile phase was propanol: water: ammonia (70:20:10% v/v), then visualization was carried out using diphenyl amine-aniline reagent [13, 60]. Additionally, the N-acetyl chitopentose hydrolysate was analyzed by high-performance liquid chromatography (HPLC) and compared to the enzyme solution without the addition of N-acetyl chitopentose. HPLC analysis was carried by applying Agilent Technology 1100 series liquid chromatograph in which a refractive index detector, Shim-pack SCR-101 N column and ultrapure water as a mobile phase adjusted at flow rate 0.7 mL min^− 1^ were used.

### Enzyme immobilization

#### Preparation of UiO-66 framework nanoparticles

UiO-66 was prepared according to a previously reported method with slight modifications [61]. Polyvinylpyrrolidone (0.20 g) was broken up into blended dissolvable solutions containing 4 mL of dimethylformamide (DMF) and 4 mL of ethanol. This was followed by the breakdown of 24.2 mg of ZrCl_4_ (0.1 mmol) and 5.43 mg of terephthalic acid (0.03 mmol) in 4 mL of DMF. After that, the mixture was ultrasonicated for 20 min. Hence, the mixture was exchanged into a Teflon-lined stainless-steel autoclave and warmed at 100 °C for 8 h. Finally, the obtained material was collected by centrifugation, water-rinsed, and dried in a vacuum solidification drier.

### Physicochemical characterization of synthesized UiO-66 framework nanoparticles

#### Transmission electron microscopy

Transmission electron microscope (TEM) imaging was used to evaluate the morphology of the UiO-66 framework nanoparticles. The samples were added to a copper-coated grid, stained with 1% phosphotungested acid then examined under JEM-2100 electron microscope; Jeol, Tokyo, Japan.

#### Scanning electron microscopy

The prepared nanoparticles were assessed under a scanning electron microscope (SEM) (Quanta 250, HRFEG, Czech), in which the particles were mounted on carbon tape and then coated with a thin gold palladium layer. Finally, the sample was subjected to photomicrographing at an acceleration voltage of 20 kV.

#### Fourier transform infrared spectroscopy analysis

The functional groups and chemical bonds of the UiO-66 framework nanoparticles were assessed by Fourier transform infrared spectroscopy (FTIR-8300, Shimadzu, Japan) according to the manufacturer’s instructions.

#### X-ray diffraction

X-ray diffraction (XRD) profile was estimated using an XRD diffractometer (PANalytical Empyrean, Switzerland) with a Cu-Kα (ʎ = 1.5405 Å) radiation source of 30 mA and 40 kV.

#### Particle size analyzer and zeta sizer

The polydispersity index of the size distribution and the particle size of the UiO-66 framework nanoparticles were estimated at ambient temperature with an angle of detection of 90 by photon correlation spectroscopy using a 90 plus particle size analyzer (Brookhaven Instruments Corporation, Holtsville, NY). The surface charge of the obtained nanoparticles was measured by photon correlation spectroscopy using a 90 plus Zetasizer (Brookhaven Instruments Corporation, Holtsville, NY).

### Enzyme loading

Immobilization of the produced chitinase was performed using the prepared nanocarrier to form Chitinase@UiO-66 by adding 2.5 mL of the enzyme (5 mg protein/mL) to 100 mg of the carrier under shaking at 180 rpm for different periods (1–24 h). After certain immobilization period, the nanocarrier was collected by centrifugation at 8000 rpm for 10 min, then washed twice with distilled water. The amount of the protein in the loading solution before and after immobilization was determined according to the method described in the enzyme activity and protein content determination section, the immobilization yield was determined according to Eq. 3.

3 $$Immobilization{\text{ }}yield{\text{ }}\left( \% \right){\text{ }} = {\text{ }}\left[ {\left( {{P_A} - {P_B}} \right)/{P_A}} \right]*100$$

where P_A_ is the protein content in the loading solution and P_B_ is the supernatant protein content after the immobilization process.

The immobilized enzyme activity was estimated as previously described after resuspending the collected nanocarrier in the buffer and consequently, its immobilization efficiency was determined according to Eq. 4.

4 $$Immobilization{\text{ }}efficiency{\text{ }}\left( \% \right){\text{ }} = {\text{ }}\left( {E/{E_A} - {E_B}} \right)*100$$

where E_A_, E_B_ and E were the loaded, un-bound and the bounded chitinase activities, respectively.

### Assessment of the stability of the immobilized chitinase

Initially, the impact of pH on the Chitinase@UiO-66 activity in comparison to that of the free chitinase was evaluated by determining the enzyme activity at different pH values ranging from 4 to 9 using 0.05 M acetate buffer (pH 4–5), phosphate buffer (pH 6–8) and Tris-HCl buffer (pH 9). After that, the stability of the was estimated at its optimum pH by the determination of the residual activity after 2 h of preincubation at that pH.

Moreover, at the optimum pH, the enzyme activity was determined at different temperatures ranging from 30 to 70 °C, and the enzyme activation energy (E_a_) was subsequently calculated on the basis of an Arrhenius plot (plotting ln the activation energy versus the reciprocal of the temperature) according to the following equation:

5 $$Slope{\text{ }} = {\text{ }}{E_a}/R$$

where R is the gas constant.

The thermal stability of the enzyme was evaluated by the determination of the residual activity after preincubation of the enzyme (without the addition of its substrate) at different temperatures for different incubation periods up to 2 h. The unincubated enzyme was considered to have 100% activity. On the basis of the observed results, the thermal inactivation kinetic parameters were calculated according to the following equations:

6 $$Slope{\text{ }}of\,the\,Arrhenius{\text{ }}plot{\text{ }} = {\text{ }} - {E_d}/R$$

7 $${T_{1/2}} = {\text{ }}ln\left( 2 \right)/{K_d}$$

where E_d_ is the decay activation constant and K_d_ is the thermal deactivation rate constant [62].

#### Recyclability evaluation

The reusability of Chitinase@UiO-66 was evaluated via the estimation of its catalytic activity in the hydrolysis of 4-nitrophenyl N-acetyl-β-D-glucosaminide as described in the enzyme activity determination. After each cycle, Chitinase@UiO-6 was collected by centrifugation at 8000 rpm for 10 min, followed by washing twice with distilled water and then reuse in a new reaction.

### Hydrolysis of chitin

Initially, the chitin catalytic activity of the free enzyme was measured using shrimp chitin at a concentration of 10 mg/mL in 50 mM acetate buffer, pH 5 via the addition of equivalent volume of the enzyme with activity of 50 U/mL and incubated at 50 °C for 30 min. The amount of reducing sugar released, was quantified by DNS [63] using N-acetyl glucosamine as a standard. In addition, the activity of the free enzyme as well as that of the immobilized form was evaluated under the optimum conditions for each form (pH 5 and 60 °C for the free enzyme and pH 8 and 60 °C for the immobilized form) using different chitin concentrations (1–40 mg/mL). On the basis of the obtained results, a Lineweaver‒Burk plot was generated, and the kinetic constants were determined according to Eq. (8).

8 $$1/V{\text{ }} = {\text{ }}\left( {1/{V_{max}}} \right){\text{ }} + {\text{ }}\left( {{K_m}/{V_{max}}} \right)\left( {1/S} \right)$$

where V is the enzyme activity (mmol/mL/h), V_max_ is the maximal activity, K_m_ is the Michaelis‒Menten constant and S is the chitin concentration (mg/mL) [64].

### **Assessment of the activity of chitinase and chitinase-loaded UiO-66 framework nanoparticles against*C. auris***

The antifungal activity was evaluated by the following minimum inhibition concentration (MIC) assay according to the modified Clinical and Laboratory Standards Institute (CLSI) [65]. The *C. auris* strain obtained from CDC was maintained in Sabouraud dextrose agar (SD) and then cultured overnight by inoculating one colony into 2 mL SD broth media then left to incubate at 37 °C. The next day, overnight cultures were incubated with either free chitinase or Chitinase@UiO-66 at 10^4^ CFU/ml in a flat-bottom 96-well plate. Free chitinase or Chitinase@UiO-66 were used at concentrations of (20, 10, 5, 2.5, 1.25, 0.625, 0.3125 U/mL). Unloaded UiO-66, untreated *C. auris* and free media were used as controls, and the plate was incubated for 48 h at 37 °C without shaking. Following the incubation period, the turbidity, which represents *Candida* growth at OD_600,_ was recorded by a microplate reader. The lowest concentration that induced a 50% reduction in *Candida* growth compared to that of the control was recorded as the MIC_50_ value. The experiment was performed in triplicate, and the average value was recorded.

### Statistical analysis

The data were collected and are displayed as the mean ± SD of the mean. GraphPad Prism 8.02 for Windows (GraphPad Inc., La Jolla, CA, USA) was used to calculate the MIC_50_ and generate the graphs for antifungal activity.

## Results

### Identification of the isolated microorganism

**Fig. 1:**
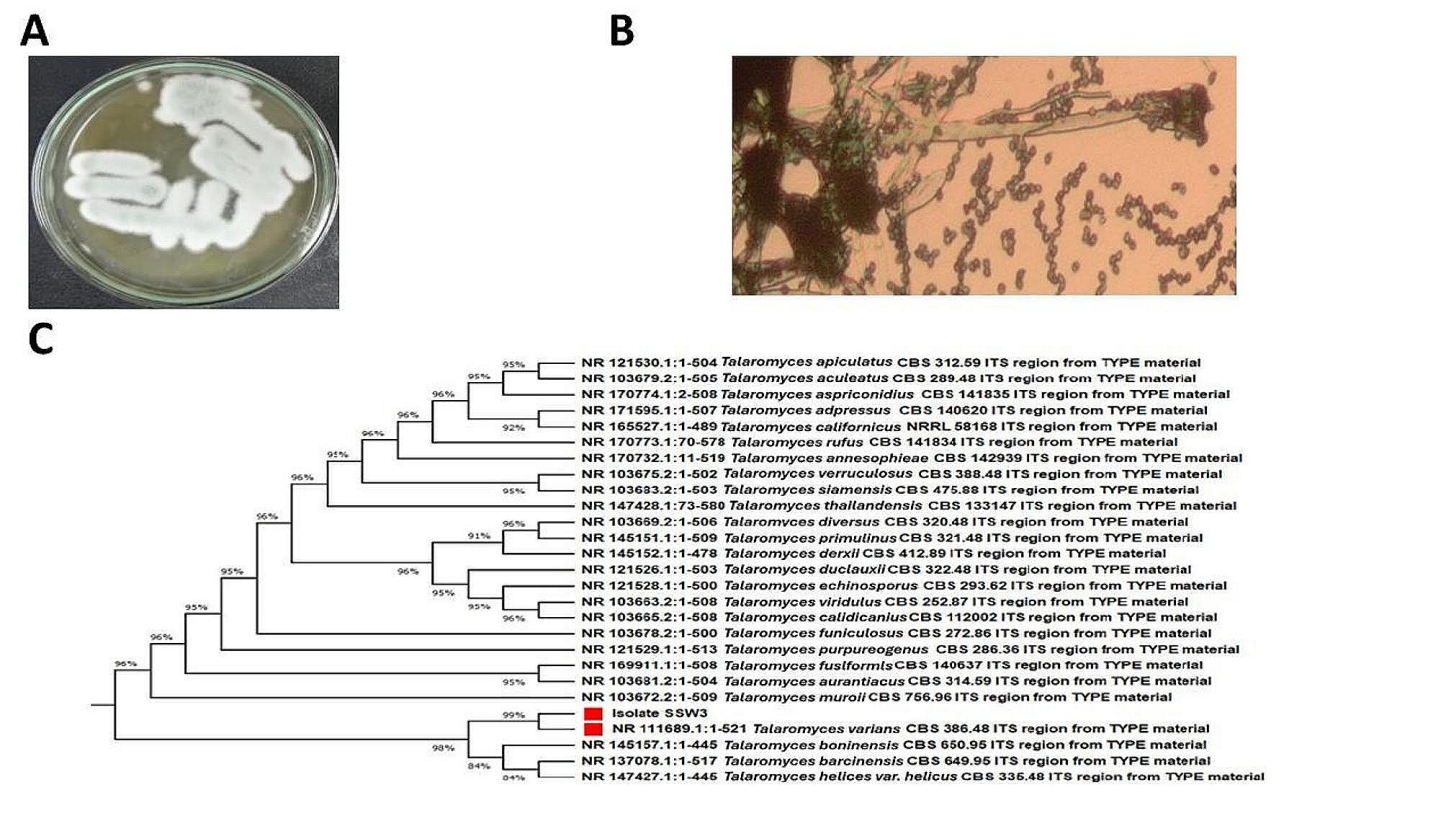
Identification of the isolated microorganism **(A)** PDA cultural features of the isolated fungus, **(B)** microscopic appearance, and **(C)** MEGA X-constructed phylogenetic tree

The isolated fungus examined in the present study showed a greenish cottony appearance on PDA (Fig. 1A) with the typical microscopic structure of the genus *Talaromyces*, as shown in Fig. (1B). Sequencing data obtained from 18 S rDNA sequencing analysis were compared to the GenBank database, which estimated the identification of the isolated strain as *Talaromyces varians.* The phylogenetic tree was constructed as illustrated in Fig. (1 C). The data was submitted to NCBI under the name *Talaromyces varians* SSW3 and acquired an accession number MW548601.

### **Production of chitinase enzyme using*Talaromyces varians*SSW3 was increased by optimizing the fermentation process**

**Fig. 2:**
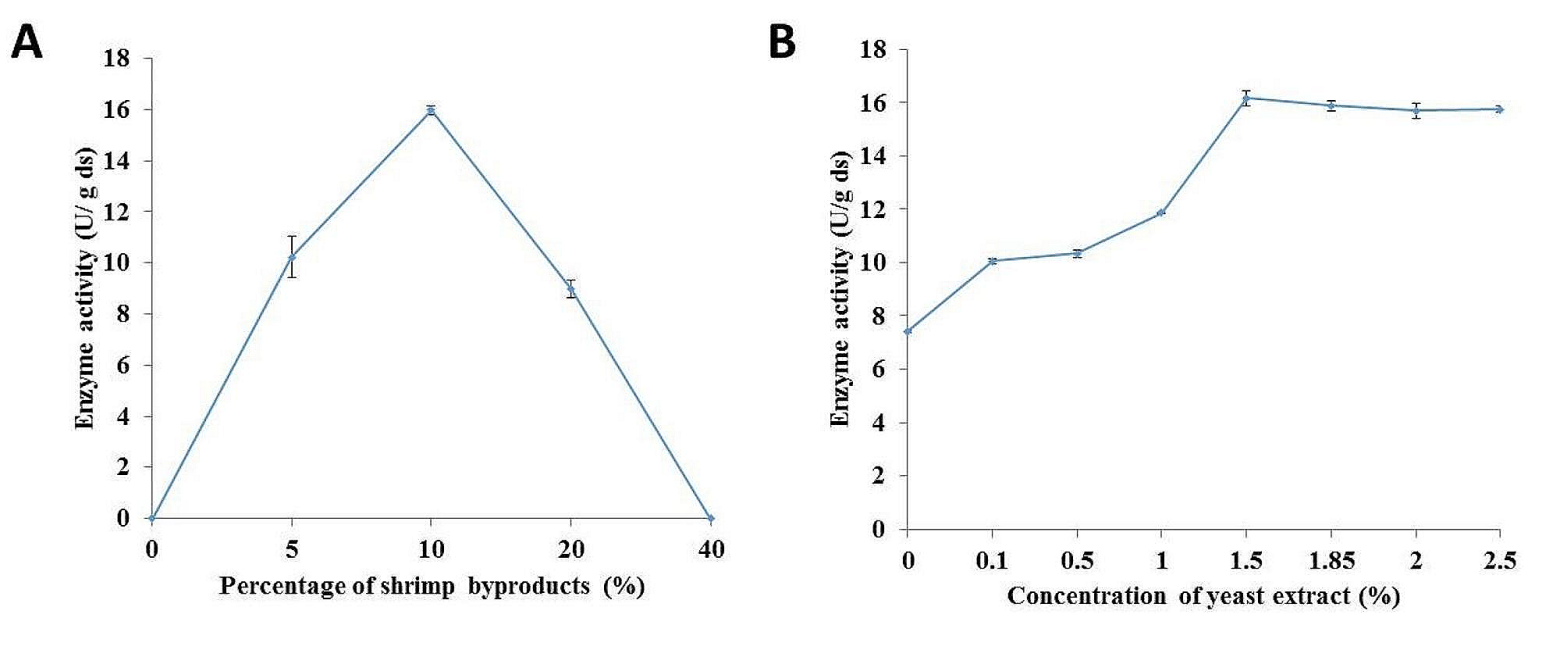
Effect of** (A) **the combination of shrimp-byproducts with wheat bran at different ratios and **(B)** the yeast extract concentration in the moistening solution on the enzyme productivity

The initial chitinase production under the described fermentation conditions was 8.97 ± 0.34 U/g dry substrate (ds). The impact of the presence of each salt in the fermentation medium was evaluated and the results estimated that the absence of MgSO_4_ and FeSO4·7H2O did not affect the enzyme productivity, therefore they were removed from the fermentation medium that subjected to further optimization process. To maximize productivity, the effects of combining shrimp-byproducts and wheat bran at different ratios were examined. The results indicated that the enzyme productivity increased as the percentage of the added shrimp-byproducts increased to 10% (w/w), with a significant decrease resulting from further increases (Fig. 2A). The effect of the yeast extract concentration in the moistening solution was also evaluated. The results indicated that the enzyme productivity was 7.42 ± 0.05 U/g ds without the addition of yeast extract and increased by 2.18-fold, reaching 16.18 ± 0.28 U/g ds by the increase of yeast extract concentration to 1.5% (Fig. 2B).

For statistical optimization, the impact of seven variables on the productivity of the enzyme was evaluated via the application of Plackett–Burman design, as shown in Supplementary Tables (1& 2). The highest activity (26.83 U/g ds) was estimated by using a moistening solution with a K_2_HPO_4_ concentration of 1.5% and a KCl concentration of 2%, and the pH was adjusted to 5 in addition to the use of a 50% (v/w) inoculum size, followed by incubation of the culture media for 5 days at 30 °C.

**Fig. 3:**
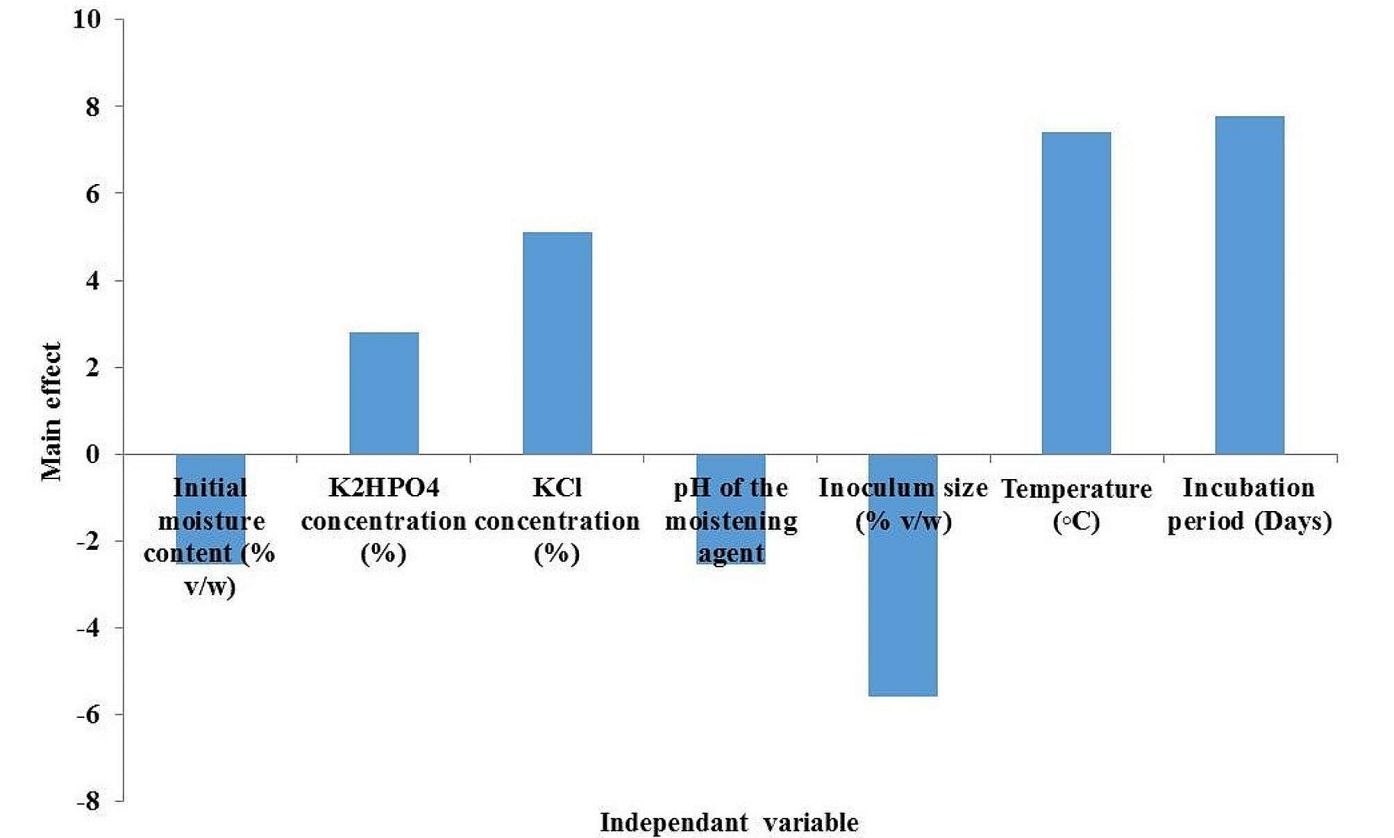
The main effect of the variables examined in the Plackett–Burman design

According to the analysis of the experimental results, the analysis of variance (ANOVA) estimated the significance of the applied model terms, as the Prob > F value was 1.38 E^− 10^. In addition, the correlation coefficient (R^2^) value was estimated to be very close to one, with a significant influence of all of the examined variables (*p* < 0.05). The core effects of the variables were recorded and represented graphically, as shown in Fig. (3). The inoculum size (A), temperature (B), and incubation period (C) were selected for further optimization because they were the variables that had the greatest influence; the inoculum size exerted a negative effect, while the others exerted positive effects. The negative effect indicated a greater influence on the examined variable when it was at the negative level (-1), while the positive effect had the opposite effect. Further optimization of the selected variables was carried out by applying the Box‒Behnken design, and the experimental results are shown in Supplementary Tables (3 & 4). ANOVA of the results estimated that the recorded *p* value for the model was < 0.0001, with an R^2^ value of 0.99, proving its significance and validity. The lack of fit was nonsignificant, indicating the fitness of the applied design, and by plotting the normal plot of residuals as shown in Figure S1 (Additional file), the plot estimated that the error terms were normally distributed since moderate scattering was observed amidst the straight line. In addition, the analysis of the data estimated that the linear, quadratic and interactive terms of the temperature and incubation period were significant. The significance of the interaction between these two variables could be determined from the steep 3D curve that was used to monitor the influence of these two variables on enzyme productivity as shown in Figure S2 (Additional file).

9 $$\begin{gathered} Chitinase{\text{ }}activity{\text{ }} = {\text{ }}26.54{\text{ }} + {\text{ }}0.2446A{\text{ }} + {\text{ }}32.1B{\text{ }} + {\text{ }} \hfill \\\,\,\,\,\,\,\,\,\,\,\,\,\,\,\,\,\,\,\,\,\,\,\,\,\,\,\,\,\,\,\,\,\,\,\,\,\,\,\,\,\,\,\,\,\,14.13C{\text{ }} + {\text{ }}1.85AB{\text{ }}--{\text{ }}2.25AC{\text{ }} + {\text{ }} \hfill \\\,\,\,\,\,\,\,\,\,\,\,\,\,\,\,\,\,\,\,\,\,\,\,\,\,\,\,\,\,\,\,\,\,\,\,\,\,\,\,\,\,\,\,\,10.5BC{\text{ }} + {\text{ }}1.75A2{\text{ }} + {\text{ }}23.72B2{\text{ }} + {\text{ }} \hfill \\\,\,\,\,\,\,\,\,\,\,\,\,\,\,\,\,\,\,\,\,\,\,\,\,\,\,\,\,\,\,\,\,\,\,\,\,\,\,\,\,\,\,\,\,\,\,\,9.86C2 \hfill \\ \end{gathered}$$

The recorded data suggested culturing the fermentation medium with an inoculum size of 20% v/w and incubating the culture medium at 35 °C for 13 days. These culture conditions led to the production of 118.77 U/g ds chitinase activity. Upon performing a confirmatory run, a chitinase activity of 120.41 U/g ds was estimated, confirming the adequacy of the model.

### ***Talaromyces varians*SSW3 chitinase exhibited promising hydrolysis activity**

**Fig. 4:**
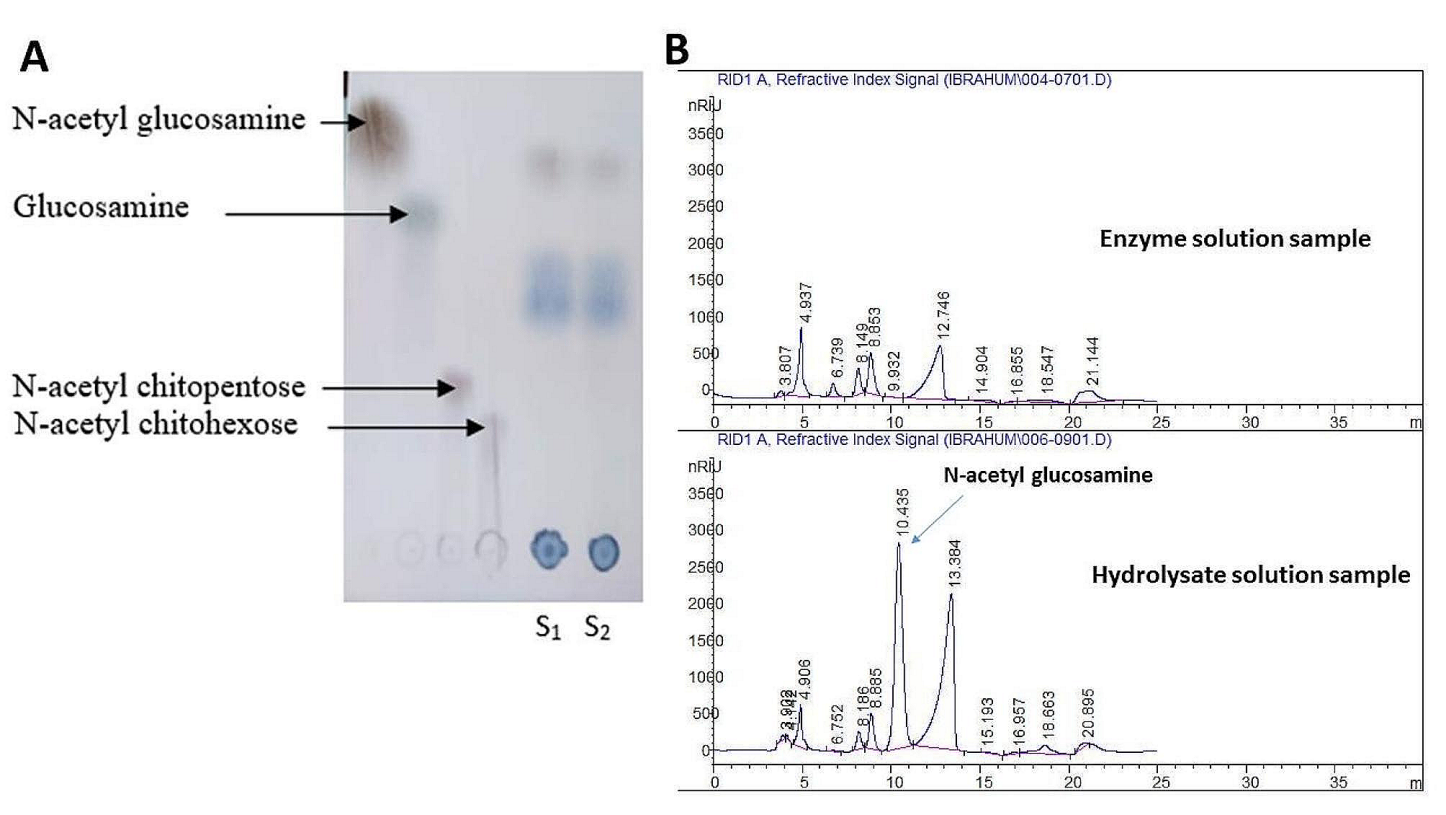
The hydrolysis activity of*Talaromyces varians*SSW3 chitinase, **(A) **TLC of the hydrolysis product, **(B)** HPLC analysis for both of the enzyme solution and the hydrolysate products

Initially, fractional precipitation using ethanol and acetone was carried out for the culture supernatant with the highest activity. The results demonstrated that the 50% acetone-precipitated fraction had a specific activity of 26.31 U/mg protein, with 76.12% recovery activity and a purification fold change of 9.11. After that, the applicability of the produced enzyme in the hydrolysis of N-acetyl chitopentose and N-acetyl chitohexose was examined, and the results indicated that the produced enzyme possessed N-acetyl-β-glucosaminidase activity, as clarified by the results of the TLC analysis (Fig. 4A). In addition, HPLC analysis of the N-acetyl chitopentose hydrolysate confirmed the hydrolysis of the oligosaccharide and the release of N-acetyl glucosamine, in which the retention times for N-acetyl chitopentose and N-acetyl glucosamine were 6.061 and 10.424 min, respectively (Fig. 4B).

### The nanoscale size of the UiO-66 framework

**Fig. 5:**
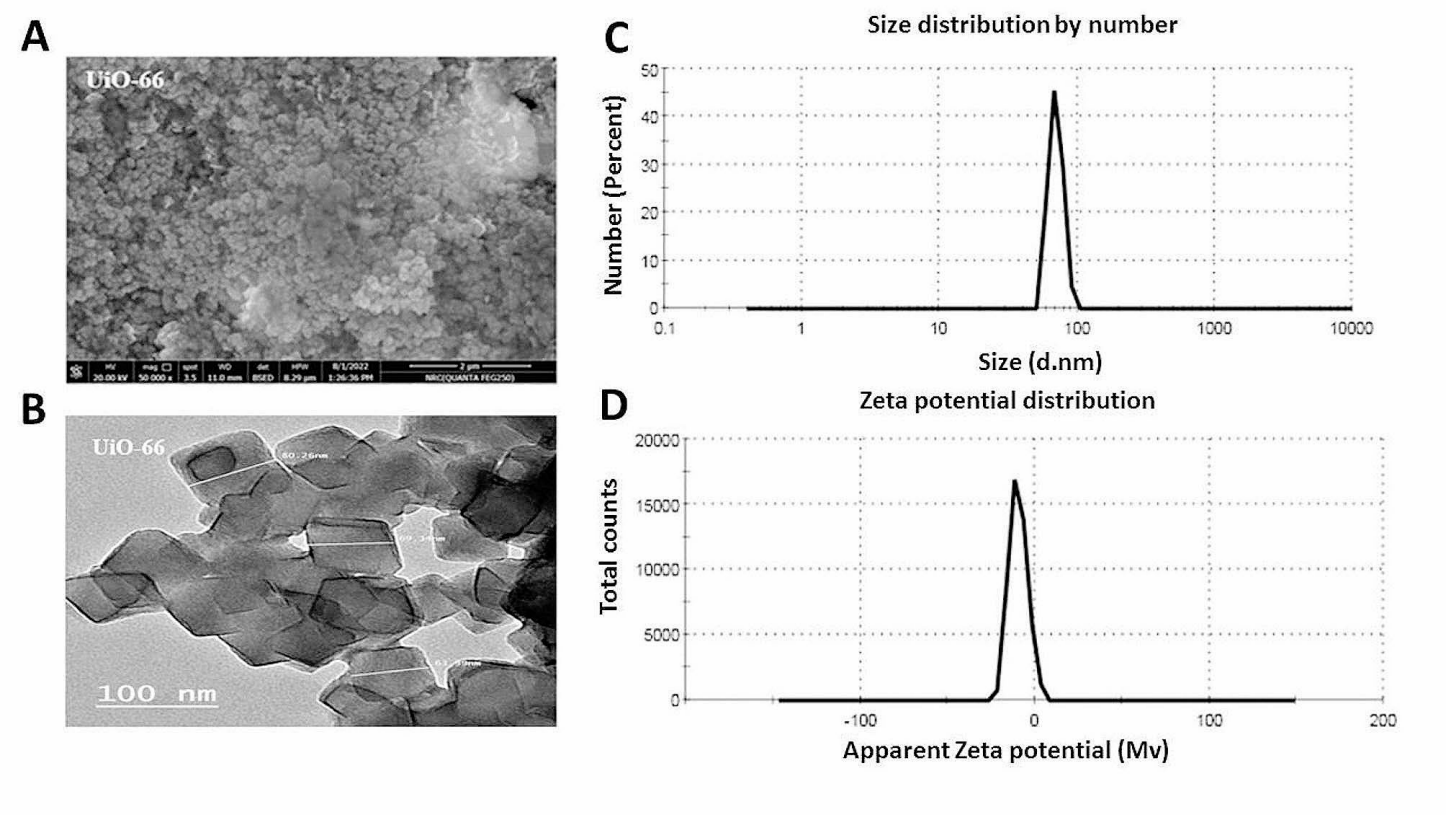
Analysis of the UiO-66 framework by **(A)** SEM, **(B)** TEM, **(C)** particle size analyzer, and **(D)** zeta sizer

The size, charge and shape of the prepared UiO-66 framework were characterized by different techniques in the present study. SEM and TEM images showed that the UiO-66 framework was cubic in shape, uniform in size, and had a smooth surface with a nanoscale particle size, as shown in Fig. 5 (A, B). The particle size analysis confirmed the SEM and TEM results, as the measured size was 70.42 ± 8.43 nm, and the PDI was 0.202 (Fig. 5C). The surface charge observed by a zeta sizer was − 9.39 ± 5.24 mV, as demonstrated in Fig. 5D.

### UiO-66 framework nanoparticles successfully immobilized chitinase and increased its stability

**Fig. 6:**
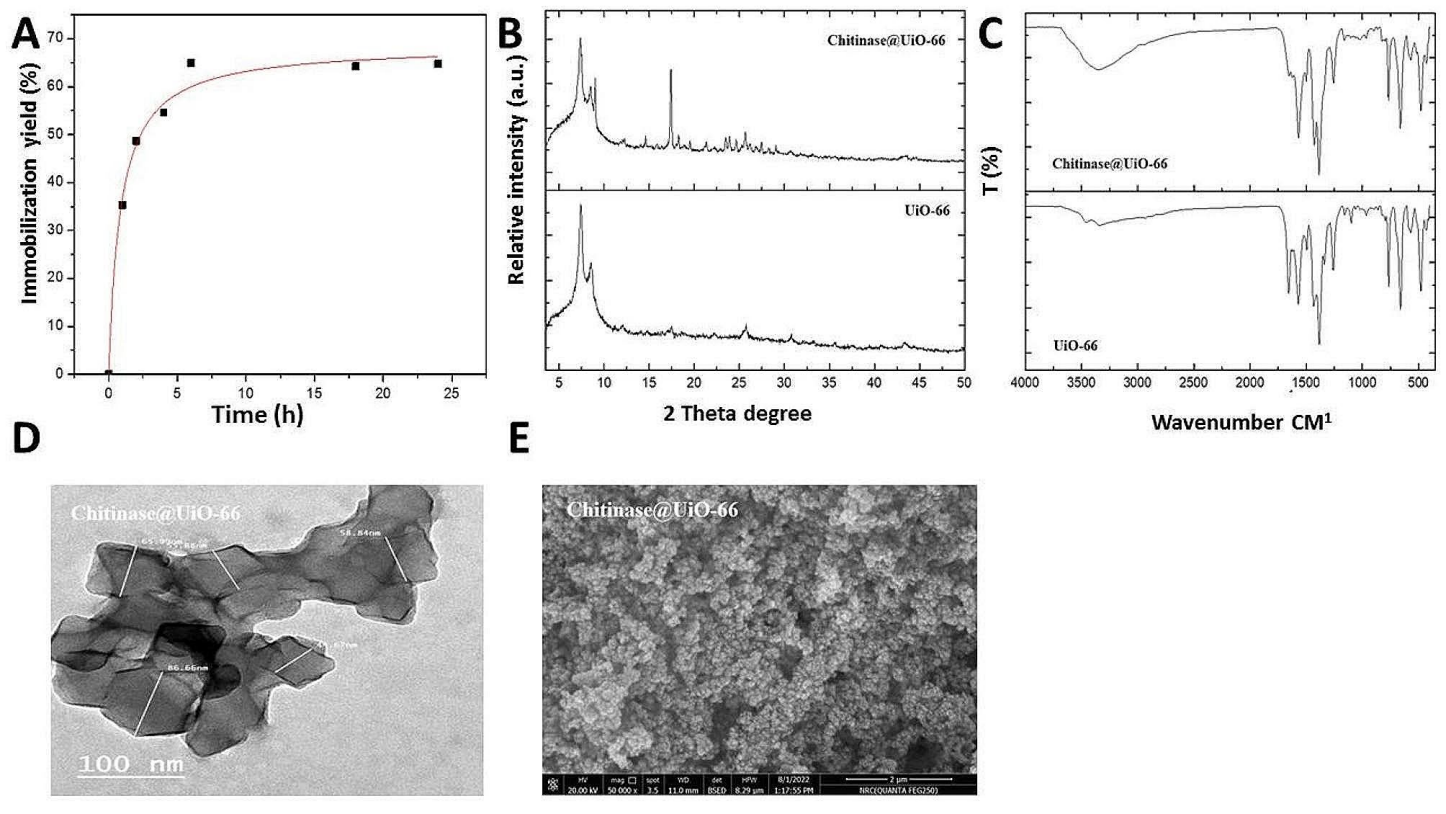
Analysis of Chitinase@UiO-66 by **(A)** immobilization yield, **(B)** XRD, **(C) **FTIR, **(D)** TEM, and **(E) **SEM

In the present study, the produced chitinase was immobilized on the prepared UiO-66 to form Chitinase@UiO-66. By determining the amount of the enzyme present in the loading solution before and after immobilization, the immobilization yield was estimated to be 36 ± 3.5% for the 1 h loading period, which increased to 65 ± 1.2% after 6 h without any significant increase thereafter (Fig. 6A). Moreover, by determining the activity of the immobilized enzyme, its immobilization efficiency was estimated to be 67 ± 2.5%.

The XRD of UiO-66 possessed the characteristic reflections of the cubic UiO-66 (ICDD PDF-4 card #71–0285), it indicated that the diffraction peaks of UiO-66 appeared sharply at 2θ values of 7.35 and 8.40 without a significant change in the position or pattern of the reflections after enzyme immobilization (Fig. 6B). The FTIR spectrum of UiO-66 exhibited distinct peaks corresponding to the stretching vibrations of C = O and C = C at 1400–1700 cm-1. The appearance of these peaks was evident for the synthesis of UiO-66. Additionally, a broad band at approximately 3000 cm‐1 was detected after the immobilization process (Fig. 6C). The morphological properties of the prepared Chitinase@UiO-66 were explored using SEM and TEM. Figure 6D and E show that the prepared UiO-66 framework nanoparticles maintained their shape after the immobilization process.

**Fig. 7:**
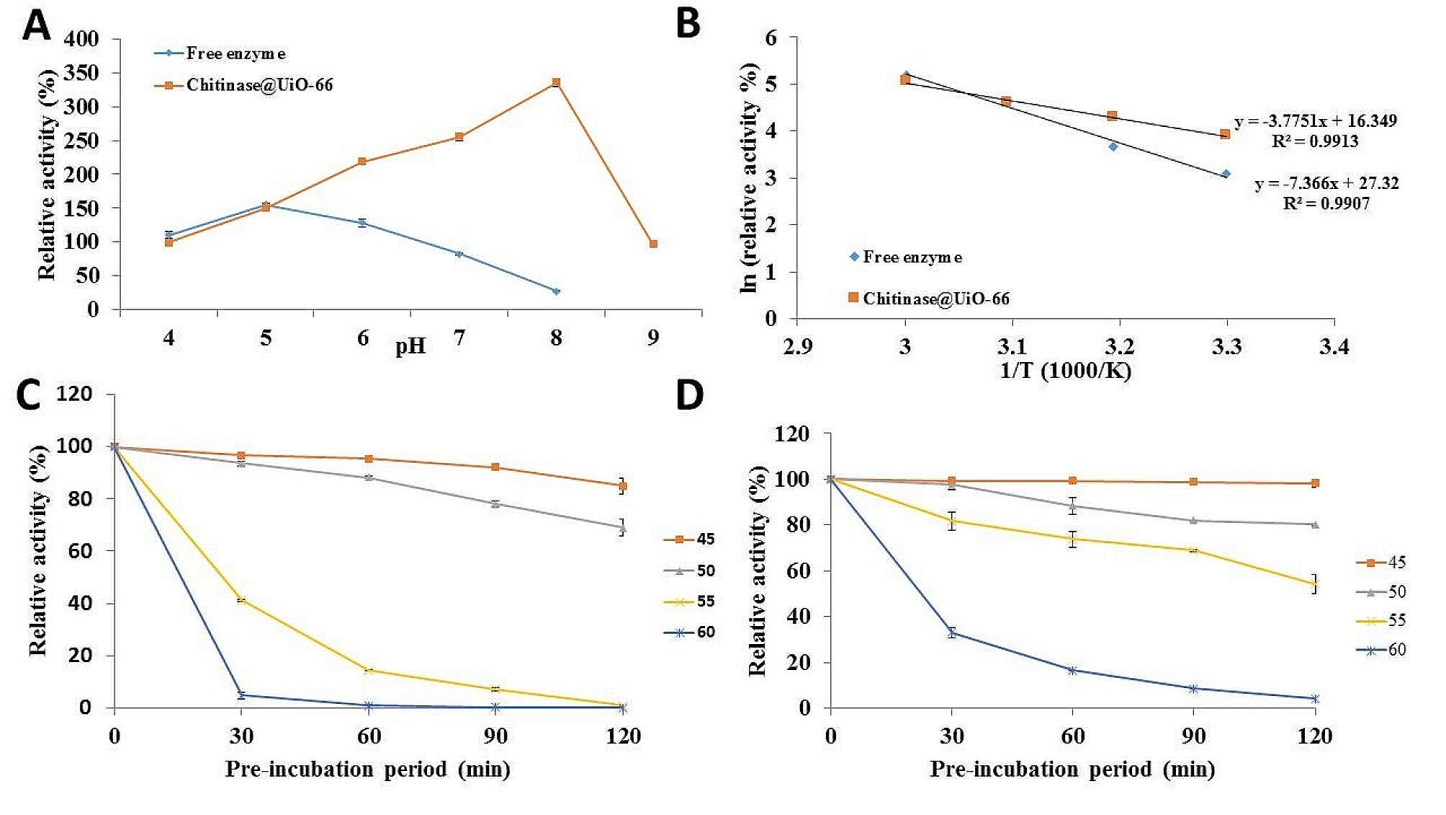
Effect of pH and temperature on chitinase and chitinase@UiO-66.** (A) **pH effect, **(B) **Arrhenius plot for thermal activation of free chitinase and chitinase@UiO-66, **(C)** thermal stability of free enzyme, **(D)** thermal stability of Chitinase@UiO-66

The effects of pH and temperature on the enzyme activity and stability before and after immobilization were evaluated. Figure 7A shows that the produced enzyme was active at pH 4–6. After immobilization, the enzyme activity improved, and the enzyme exhibited activity over a broad range of pH values (4–9) with complete stability at pH values exceeding 2 h. By plotting an Arrhenius plot (Fig. 7B), the activation energy for the immobilized enzyme was calculated to be 31.39 ± 1.41 KJmol-1, which was approximately half that of the free enzyme (61.24 ± 1.79 KJmol-1). Our thermal stability data resulted by determining the residual activity of the enzyme after pre-incubation at different temperatures ranged from 45 to 60 °C up to 2 h, showed that the immobilized enzyme had greater thermal stability than the free enzyme, as shown in Fig. 7 (C, D). Consequently, the calculated K_d_ and T_1/2_ values illustrated in Table 1 indicated a lower denaturation rate for the immobilized enzyme since it exhibited lower k_d_ and higher T_1/2_ values than the free enzyme. For the immobilized enzyme, lower k_d_ and higher T_1/2_ values were estimated to be approximately 12-, 2-, 8- and 2-fold greater at 45, 50, 55 and 60 °C, respectively, than those of the free enzyme. Moreover, the recyclability evaluation of the immobilized enzyme indicated its ability to maintain complete activity for more than 10 cycles.

**Table 1:** Thermokinetic parameters of *Talaromyces varians* SSW3 chitinase and chitinase@UiO-66

	Temperature (^ο^C)	K_d_ (min^− 1^)	T_1/2_ (min)
Free enzyme	**45**	1.2*10^− 3^	577.62
	**50**	3.1*10^− 3^	223.6
	**55**	35*10^− 3^	19.8
	**60**	45*10^− 3^	12.84
Chitinase@UiO-66	**45**	0.1*10^− 3^	6931.47
	**50**	2*10^− 3^	346.57
	**55**	4.6*10^− 3^	150.68
	**60**	25.6*10^− 3^	27.08

### The hydrolytic activity of chitinase was enhanced after immobilization on UiO-66 framework nanoparticles

**Fig. 8:**
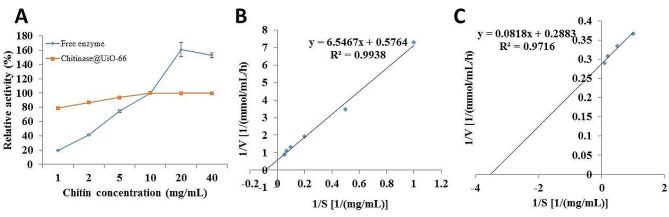
**(A)** Effect of chitin concentration on the activity of free chitinase and chitinase@UiO-66 in addition to the Lineweaver‒Burk plot for **(B)** free chitinase and **(C) **chitinase@UiO-66

The chitin catalytic activity of the free enzyme was initially estimated to be 0.27 U/mg protein. Moreover, the enzyme activity was evaluated using different chitin concentrations, and the results shown in Fig. (8 A) indicate that the enzyme activity increased with increasing the concentration up to 20 mg/mL for the free form compared to 10 mg/mL for the immobilized form. Consequently, the K_m_ values calculated on the basis of the Lineweaver‒Burk plot were 11.11 ± 0.79 and 0.29 ± 0.01 mg/mL for the free and immobilized enzymes, respectively. In addition, the calculated V_max_ values were 1.74 ± 0.19 and 3.47 ± 0.03 mmol/mL/h for the free and immobilized enzymes, respectively, as illustrated in Fig. 8 (B, C). The increase in the V_max_ and decrease in the K_m_ of the immobilized enzyme were estimated to be approximately 2- and 38-fold greater, respectively, than those of the free enzyme.

### **Loading chitinase on UiO-66 framework nanoparticles increased its antifungal activity against*C. auris***

**Fig. 9:**
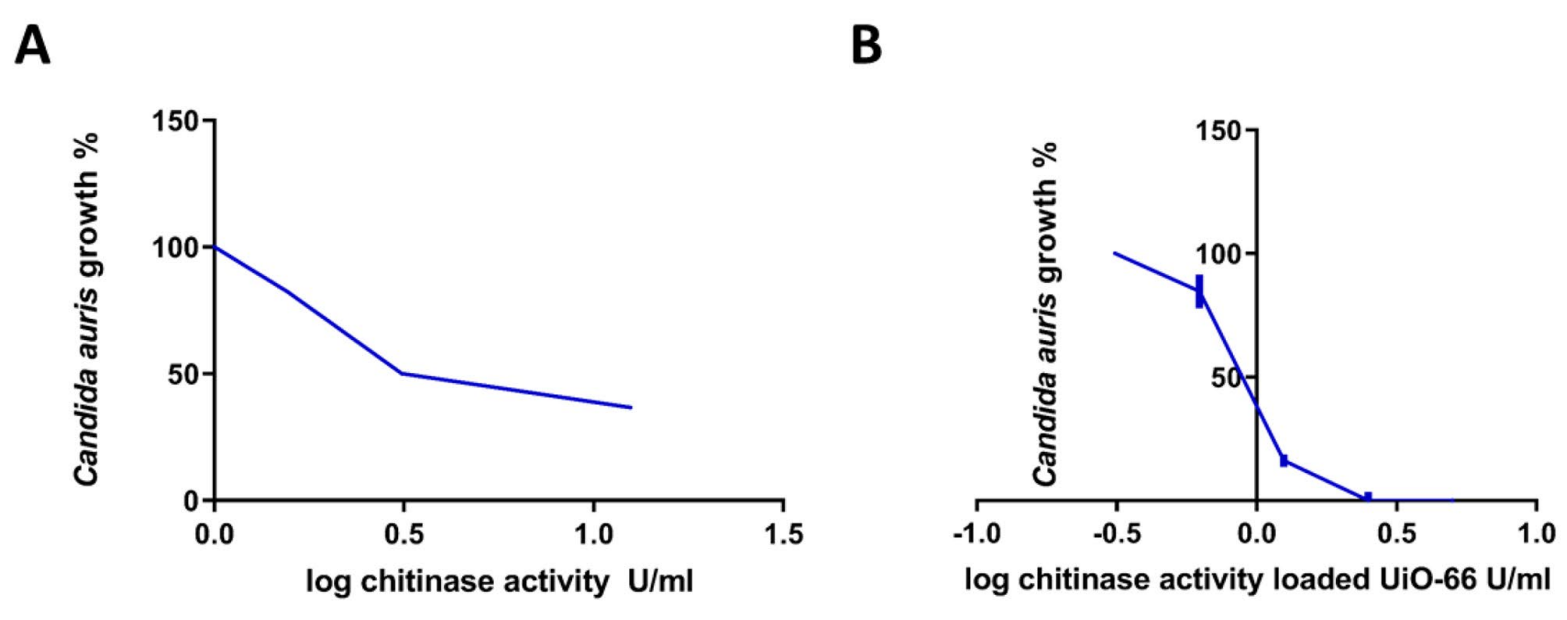
Antifungal activity of **(A)** free chitinase and **(B)** chitinase@UiO-66 against *C. auris*

Initially, the activity of chitinase against *C. auris* was evaluated, and its MIC_50_ was found to be 5.582 ± 0.57 U/mL. Interestingly, the activity of chitinase was improved more than 6-fold following loading on UiO-66 framework nanoparticles, as the determined MIC_50_ decreased to 0.89 ± 0.056 U/mL, as shown in Fig. 9.

## Discussion

Food-processing byproducts have attracted growing interest as economic substrates for the production of various microbial enzymes [54, 66, 67]. Chitin constitutes 20 to 30% of crustacean shells [68]. In the current study, shrimp-byproducts were used for inducing the fungal production of chitinase under SSF. Chitinases generally play multiple physiological roles in fungi, so they contribute a large share of all chitinase-producing microbes [22]. Herein, a chitinase-producing fungus that was subsequently identified as *Talaromyces varians* was isolated. Benjamin in 1955 described the *Talaromyces* genus as a *Penicillium* sexual state that produces soft ascocarps covered by interwoven hyphae [69]. Generally, some *Talaromyces* species are used to produce valuable products with various biotechnological applications. For instance, species of *Talaromyces* were found to produce rugulosin [69]. Rugulosin has antibacterial activity against *Staphylococcus aureus* and the parasitic oomycete Pythium [70, 71]. Additionally, they can produce mitorubrins and Monascus pigments that have several industrial and biotechnological applications [72]. Furthermore, *Talaromyces flavus* is employed as a biocontrol agent for soil-borne pathogens [69]. On the other hand, the ability of *Talaromyces* to produce industrially important enzymes such as β-rutinosidase, phosphatase, endoglucanase, and cellulase was confirmed by several studies [73–75]. This encouraged us to explore the ability of the isolated strain to ferment shrimp-byproducts and to produce chitinase via the SSF process. In the last few years, shrimp-byproducts have been used for inducing the production of chitinolytic enzymes instead the using of the refined substrate (chitin) from the perspective of cost reduction in addition to environmental protection [76, 77]. In addition, agro-industrial wastes, including wheat bran, are porous biomaterials amenable for the growth of microorganisms in addition to having the capacity to hold the requisite moisture for SSF. Therefore, wheat bran supplemented with chitin or chitin-rich sources under SSF has been widely used to induce the production of chitinolytic enzymes [19, 78, 79].

We also evaluated the ability of yeast extract to enhance enzyme productivity. Notably, examining the variation in either the concentration of shrimp byproduct or the concentration of yeast extract increased the enzyme productivity more than 2-fold in the present study. This result agrees with that of Ismail et al. [54], who reported that the addition of yeast extract to moistening agents improved enzyme productivity. To further enhance enzyme productivity, statistical optimization via the application of Plackett‒Burman and Box‒Behnken methods was performed. Plackett–Burman and Box–Behnken designs are commonly used to make experimentation more precise and efficient. Both mathematical and graphical models are combined to determine the optimal combination of variables that yield the optimum results [19, 80]. Apart from achieving the best results, these designs reduce the required number of experiments and consequently mitigate the research cost [80]. In general, Plackett–Burman design screens various factors to specify the main factors controlling the response of interest, while Box–Behnken design considers the main effects and second-order interactions to carry out detailed exploration of the response surface. This approach can identify the complex interactions between the studied variables [81]. Statistical optimization has been extensively applied to optimize the conditions required for various biotechnological processes, including the microbial production of chitinases [82–84]. In the current study, statistical optimization revealed that the produced chitinase activity was 13.4-fold greater than the initial activity and greater than many previously reported fungal chitinase activities. For instance, Alves and his colleagues produced a chitinase enzyme from *Aspergillus niveus* with a maximum activity of 2.9 U/mL [85]. Moreover, a chitinase activity of 2.65 U/mL was estimated by Ismail et al. [54] for *Alternaria* species. Next, we confirmed that the produced chitinase enzyme acheived N-acetyl-β-glucosaminidase activity by releasing N-acetyl glucosamine. Duo-Chuan indicated that N-acetyl-β-glucosaminidases are capable of producing N-acetyl glucosamine via the hydrolysis of chitin from the nonreducing end [14]. In an effort to enhance the enzyme stability and efficiency, the produced chitinase was immobilized on UiO-66 framework nanoparticles. The immobilization of chitinase can protect the enzyme from extreme surrounding conditions and consequently improve its industrial application [86]. UiO-66 nanoparticles are metal-organic frameworks fabricated by coordination bonding between metal ion clusters and functional organic ligands [87]. UiO-66 has been estimated to be an efficient nanocarrier for the delivery of various bioactive compounds [88–91] in addition to the immobilization of some enzymes, including lipases [92], peroxidases, laccases and cellulases [93]. The immobilization of the lipase enzyme on UiO-66 improved its thermal stability at different temperatures up to 70 °C [86]. Similar findings were reported by Chen and his colleagues [92]. The immobilization of peroxidase, laccase and cellulose on UiO-66 enhanced their stabilities, as indicated by the proper fit between the UiO-66 nanoparticles and the enzyme molecular size [93]. To our knowledge, for the first time, we explored the potential of UiO-66 nanoparticles to efficiently immobilize the produced chitinase. Interestingly, loading the chitinase enzyme on UiO-66 framework nanoparticles improved its activity over a wide range of pH values, with a significant decrease in the enzyme activation energy (approximately half). The decrease in the activation energy indicated that the amount of the required energy for the formation of the activated enzyme-substrate complex was reduced, indicating its enhanced catalytic activity [94]. Moreover, an improvement in the thermal stability of the immobilized enzyme was estimated compared to the free form, as indicated by the decrease in K_d_ and increase in the T_1/2_ values. Previous studies have shown that the UiO-66 framework is considered one of the most stable MOFs [95, 96]. It can maintain its structure, framework, and topology under various chemical and thermal conditions due to its mechanical and thermal stability [95, 96]. Most synthesized enzyme-framework complexes are based on the mechanisms of inclusion, covalent bonding, trapping, or physical adsorption, resulting in strong and stable composites that can explain the improvement observed in enzyme stability after immobilization on UiO-66 framework nanoparticles [97–100]. To our knowledge, we are the first to evaluate the potential detrimental effects of chitinase and chitinase@UiO-66 on *C. auris*. The numerous outbreaks provoked by *C. auris* infection accompanied by a mortality rate of 30 to 60% encouraged researchers to decipher the resistance mechanism of *C. auris* to antifungal drugs [32, 101–105]. It is beyond the argument that chitin content plays a primary role in resistance development. Fayed et al. demonstrated that caspofungin resistance in *C. auris* mainly depends on the chitin content in the yeast cell wall [32]. Another study revealed that *C. auris* clinical isolates that exhibited resistance to fluconazole exhibited elevated levels of cell wall-chitin [102]. The significant correlation between the cell wall-chitin level and sterol biosynthesis in addition to the regulation of chitin synthesis can explain the principal role of the chitin level in the emergence of *C. auris* resistance [106, 107]. All these merits likely account for the observed effect of chitinase on *C. auris* viability in the present study. On the other hand, the differential increase in antifungal activity upon chitinase immobilization on UiO-66 could be related to the observed increase in enzyme catalytic activity after immobilization on UiO-66. The improvement in the enzymatic activity can be attributed to the decrease in the activation energy as well as the increase in the enzyme-chitin affinity estimated by the decrease in the K_m_ value. The observed reduction in the K_m_ value can indicate that chitinase requires a low chitin concentration to reach its V_max_ [108]. The major limitation of this work lies in the lack of precise validation regarding the mechanism by which UiO-66 enhances the antifungal activity of the chitinase enzyme. Hence, in future work, we will study the uptake of UiO-66 framework nanoparticles by *C. auris* by confocal microscope. We will also study the potential oxidative stress induced by UiO-66 framework nanoparticles on *C. auris* by direct measurement of intracellular reactive oxygen species. This will provide more insight into the mechanism by which UiO-66 framework nanoparticles enhance chitinase activity against *C. auris*.

## Conclusion

In this study, we investigated chitinase immobilized with UiO-66 as a potent antifungal against *C. auris*. The chitinase’s productivity improved through statistical optimization. The synthesized UiO-66 underwent comprehensive characterization, revealing a uniform cubic shape with a nano size range. Chitinase was successfully immobilized on UiO-66, notably enhancing enzyme activity and stability. Post-immobilization, the hydrolytic activity of chitinase surged, leading to an increase in Vmax and a decrease in Km. The antifungal efficacy against *C. auris* was significantly enhanced compared to the free enzyme. This study offers a promising alternative for combatting *C. auris*, highlighting the potential of chitinase immobilized with UiO-66 nanoparticles for antifungal therapy.

### Electronic supplementary material

Below is the link to the electronic supplementary material.

Supplementary Material 1

## Data Availability

Sequencing data obtained from 18 S rDNA sequencing analysis was submitted to NCBI under the name Talaromyces varians SSW3 and acquired an accession number MW548601.
