# Supplementary material for: Chitinase-functionalized UiO-66 framework nanoparticles active against multidrug-resistant Candida Auris
Source: BMC Microbiol. 2024 Jul 20;24:269. doi: 10.1186/s12866-024-03414-1 (PMC11264975; doi:10.1186/s12866-024-03414-1)
Supplement: Supplementary file 1 — Supplementary Material 1 [file 12866_2024_3414_MOESM1_ESM.pdf]

Table (1): Plackett Burman design

Run number	Initial moisture content (%v/w)	K_2HPO_4 (%)	KCl (%)	pH of the moistening agent	Inoculum size (%v/w)	Temperature (°C)	Incubation period (days)	Enzyme activity (U/g ds)
**1**	(0) -	(0.15) -	(0.2) -	(7) +	(100) +	(30) +	(5) -	10.769
**2**	(100) +	(0.15) -	(0.2) -	(5) -	(50) -	(30) +	(9) +	24.151
**3**	(0) -	(1.5) +	(0.2) -	(5) -	(100) +	(25) -	(9) +	16.483
**4**	(100) +	(1.5) +	(0.2) -	(7) +	(50) -	(25) -	(5) -	9.238
**5**	(0) -	(0.15) -	(2) +	(7) +	(50) -	(25) -	(9) +	21.839
**6**	(100) +	(0.15) -	(2) +	(5) -	(100) +	(25) -	(5) -	8.477
**7**	(0) -	(1.5) +	(2) +	(5) -	(50) -	(30) +	(5) -	26.833
**8**	(100) +	(1.5) +	(2) +	(7) +	(100) +	(30) +	(9) +	23.959

Table (2): Analysis of Plackett Burman design

	Enzyme activity analysis			
	**Coefficient**	**Standard error**	***t*-statistics**	**P-value**
**Initial moisture content (%v/w)**	-0.02525	0.001944	-12.9843	$1.17E^{-06}$
**K_2HPO_4 (%)**	2.088307	0.144029	14.49919	$5.01E^{-07}$
**KCl (%)**	2.842624	0.108022	26.31524	$4.67E^{-09}$
**pH of the moistening agent**	-1.26732	0.09722	-13.0357	$1.14E^{-06}$
**Inoculum size (%v/w)**	-0.11186	0.003889	-28.7647	$2.31E^{-09}$
**Temperature (°C)**	1.483844	0.038888	38.15697	$2.44E^{-10}$
**Incubation period (days)**	1.944669	0.04861	40.00563	$1.68E^{-10}$
**Model summary**				
**Multiple R**	0.99922			
**R^2**	0.998441			
**Adjusted R^2**	0.997078			
**Standard Error**	0.388879			

Table (3): Box-Behnken Design

Run number	Independent variable			Enzyme activity (U/g ds)
	Inoculum size (%v/w)	Temperature (°C)	Incubation period (days)	
**1**	- (20)	- (25)	0 (9)	21.260
**2**	+ (80)	- (25)	0 (9)	19.690
**3**	- (20)	+ (35)	0 (9)	80.639
**4**	+ (80)	+ (35)	0 (9)	86.457
**5**	- (20)	0 (30)	- (5)	25.316
**6**	+ (80)	0 (30)	- (5)	28.668
**7**	- (20)	0 (30)	+ (13)	52.123
**8**	+ (80)	0 (30)	+ (13)	46.479
**9**	0 (50)	- (25)	- (5)	20.838
**10**	0 (50)	+ (35)	- (5)	65.177
**11**	0 (50)	- (25)	+ (13)	34.058
**12**	0 (50)	+ (35)	+ (13)	120.409
**13**	0 (50)	0 (30)	0 (9)	29.289
**14**	0 (50)	0 (30)	0 (9)	25.757
**15**	0 (50)	0 (30)	0 (9)	24.563

Table (4): Analysis of Box-Behnken Design

Source	Sum of Squares	Df	Mean Square	F-value	P-value
**Model**	12638.87	9	1404.32	77.15	< 0.0001
**A**	0.4787	1	0.4787	0.0263	0.8775
**B**	8245.57	1	8245.57	452.99	< 0.0001
**C**	1598.11	1	1598.11	87.8	0.0002
**AB**	13.64	1	13.64	0.7494	0.4262
**AC**	20.23	1	20.23	1.11	0.34
**BC**	441.25	1	441.25	24.24	0.0044
**A^2**	11.31	1	11.31	0.6216	0.4662
**B^2**	2078.24	1	2078.24	114.17	0.0001
**C^2**	358.94	1	358.94	19.72	0.0068
**Lack of Fit**	78.93	3	26.31	4.35	0.1924
**Pure Error**	12.08	2	6.04		
**Cor Total**	12729.88	14			

df Degrees of freedom.

A Inoculum size.

B Temperature.

C Incubation period.

P-value < 0.05 was considered significant

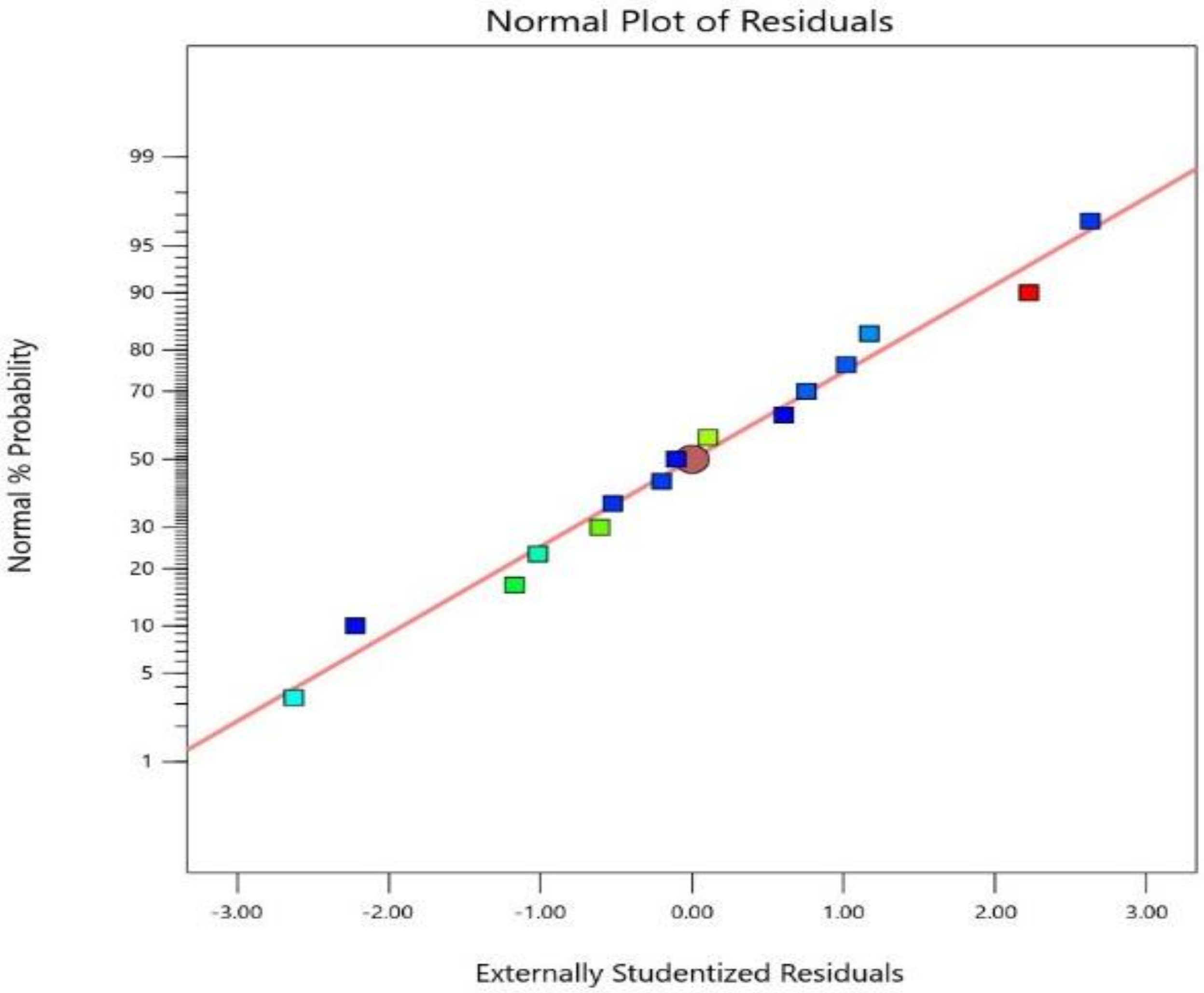

Figure S1: Normal plot of residuals.

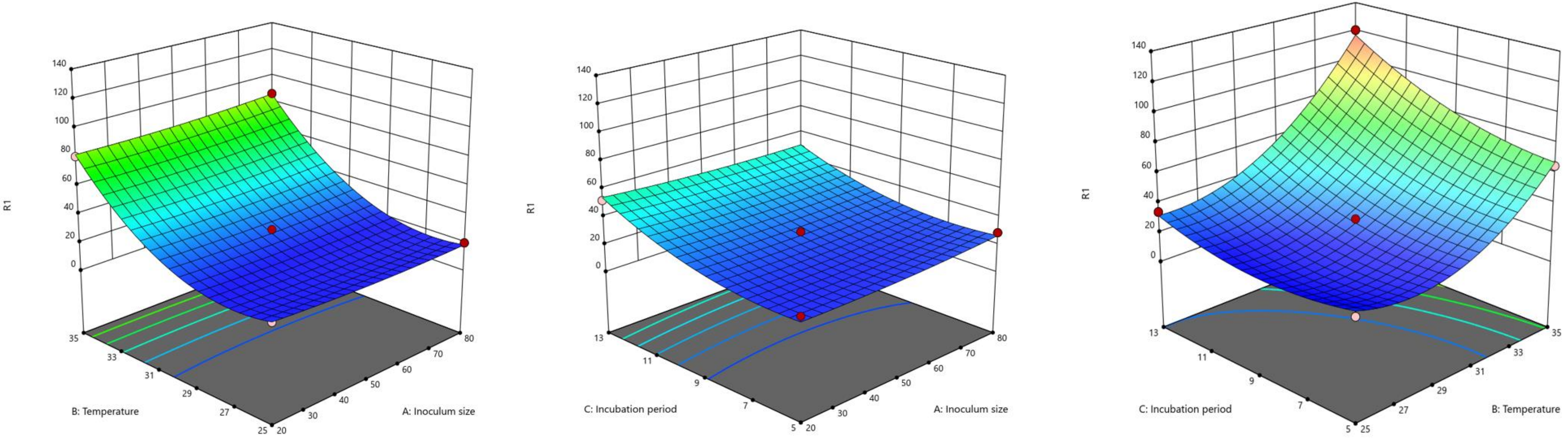

Figure S2: 3D plots monitoring the impact of modifying the examined variables in which the obtained chitinase activity was the response (R1). Each plot monitored two variables and kept the third fixed at its central level.
